# Current Implementation of Digital Dentistry for Removable Prosthodontics in US Dental Schools

**DOI:** 10.1155/2022/7331185

**Published:** 2022-04-15

**Authors:** Yoshiki Ishida, Yukinori Kuwajima, Takuya Kobayashi, Yu Yonezawa, Derek Asack, Manavi Nagai, Hisatomo Kondo, Shigemi Ishikawa-Nagai, John Da Silva, Sang J. Lee

**Affiliations:** ^1^Department of Oral Medicine, Infection and Immunity, Harvard School of Dental Medicine, 188 Longwood Avenue, Boston, MA 02115, USA; ^2^Department of Dental Materials Science, School of Life Dentistry at Tokyo, The Nippon Dental University, 1-9-20 Fujimi, Chiyoda-ku, Tokyo 102-8159, Japan; ^3^Department of Orthodontics, Iwate Medical University, School of Dental Medicine, 1-3-27 Chuo-dori, Morioka, Iwate 020-8505, Japan; ^4^Department of Prosthodontics and Dental Implantology, Iwate Medical University, School of Dental Medicine, 1-3-27 Chuo-dori, Morioka, Iwate 020-8505, Japan; ^5^DMD Class 2022, Goldman School of Dental Boston University, 635 Albany Street, Boston, MA 02118, USA; ^6^Department of Restorative Dentistry and Biomaterial Sciences, Harvard School of Dental Medicine, 188 Longwood Avenue, Boston, MA 02115, USA

## Abstract

**Objectives:**

Although digital technology has been widely integrated into dental education, there is limited literature investigating the extent of the integration of computer-aided design and computer-aided manufacturing (CAD-CAM) for removable systems in the dental curriculum. The purpose of this study was to assess the current implementation of CAD-CAM complete and partial dentures in predoctoral (PP) and advanced graduate prosthodontic (AGP) education in US dental schools. The study also aimed to identify potential barriers to its implementation in the dental curriculum.

**Methods:**

An online survey with 15 questions was created using online survey software. The survey was distributed to the directors of predoctoral prosthodontics in 56 schools and advanced graduate programs of prosthodontics in 52 schools listed in the 2018–19 American Dental Education Association (ADEA) Directory.

**Results:**

The percentage of programs (PP and AGP) implementing CAD-CAM complete dentures (CAD-CAM CDs) and CAD-CAM removable partial dentures (CAD-CAM RPDs) in their didactic, preclinical, and clinical curricula was recorded. CAD-CAM CDs are taught in didactic courses in 54.2% of PP and 65.2% of AGP. However, CAD-CAM RPDs are only taught in 37.5% of PP and 47.8% of AGP. Programs are largely limited by a lack of funds, resources, time, and faculty members.

**Conclusion:**

While digital technologies have indeed become more prevalent in dental education, many institutions face barriers to implementation. More research must be conducted in order to support the continued incorporation of digital technologies into dental education.

## 1. Introduction

Over the last decade, digital technologies such as computer-aided design and computer-aided manufacturing (CAD-CAM), intraoral optical scanners (IOSs), 3D radiographic imaging with 3D analytic software applications, and 3D printing have revolutionized dentistry. These systems have transformed the conventional clinical workflow and improved the efficiency and predictability of treatment outcomes. For example, studies have demonstrated fully digital workflows in fixed prosthodontics [1,2]. These technologies have also streamlined dental implant therapy in all phases of diagnosis, planning, guided surgical placement, and restoration of dental implants. Beyond the clinical applications, surgical simulation systems are beneficial for education and training [3–7].

The use of CAD-CAM technology in the fabrication of removable prostheses was first described in the literature in 1994 [8]. CAD-CAM technology has been utilized in removable prosthodontics since Goodacre introduced the CAD-CAM complete denture system in 2012 [4]. However, CAD-CAM technologies have not been as widely implemented in removable prosthodontics when compared with fixed prosthodontics. The limitations in removable prosthodontics may be due to difficulties in digital scanning of soft tissue and the complexity of recording maxillary-mandibular-relationships. Although only two systems were available in 2012, Dentca® (Dentca Inc., Los Angeles, CA, USA) [6] and AvaDent® (Global Dental Science, Scottsdale, AZ, USA) [7], there are more than 4 systems available today [8–11].

There are many benefits to utilizing a CAD-CAM removable prosthodontics system, both in partial and complete dentures. From a clinical standpoint, the use of CAD-CAM technology allows for digital memory and ease of manipulation. Unlike physical models, CAD-CAM technology significantly improves the efficacy and reproducibility of the denture fabrication workflow. In addition, the process is further simplified by reducing laboratory procedures. The collaboration and communication between clinicians and laboratory technicians are made easier by having a digital workflow. But how does the accuracy and precision of CAD-CAM dentures compare with traditional methods? Studies have shown that CAD-CAM complete denture (CAD-CAM CD) systems have comparable or superior clinical accuracy to that of traditional methods [12]. It has also been shown that frameworks of partial dentures produced by CAD-CAM were of clinically acceptable accuracy [13]. With the rapid advancement of technology, CAD-CAM will only continue to improve in the coming years. In addition to the clinical benefits of CAD-CAM denture systems, the quality of education is improved through digital technology. Incorporating a digital workflow allows for a more interactive and intuitive learning experience for students, especially given that the current and incoming cohorts of dental students are of a generation that has an intimate relationship with technology.

In 2014, Brownstein et al. reported that 76% of dental schools in the United States (US) taught CAD-CAM indirect restorations as part of their preclinical didactic coursework and 58% offered clinical experience with CAD-CAM indirect restorations [14]. Schweye et al. reported that 94% of students in Germany who participated in a digital dentistry curriculum showed considerable interest, and it was found that students using CAD-CAM technology prepared more teeth than their fellow classmates who did not use CAD-CAM technology [15]. A previous study evaluated the outcome of repetitive tooth preparation and the influence of intraoral scanning combined with software analysis on the acquisition of motor skills in dental students. They concluded that intraoral scanning technology was beneficial for their self-evaluation by visual feedback [16]. In 2013, Bidra et al. reported that due to the increase in technological advancements, a new host of literature related to computer-aided technology for complete dentures is emerging [17]. The study also predicted that the rising tide of complete denture CAD-CAM technology would have a significant effect on dental education, patient care, research, and public health [17]. Fernandez et al. conducted an online survey in 2014 regarding complete denture fabrication using CAD-CAM technology and concluded that the majority of respondents indicated that they plan to add digital denture fabrication to their curricula within the next 1 to 4 years [18].

Even though digital technology has been widely integrated into dental education, there is limited literature on the understanding of the integration of CAD-CAM removable systems in the dental curriculum. The purpose of this study was to assess the current implementation of CAD-CAM complete and partial dentures in predoctoral (PP) and advanced graduate prosthodontic (AGP) education in US dental schools. The study also aimed to identify potential barriers to its implementation in the dental curriculum.

## 2. Materials and Methods

**Figure 1:**
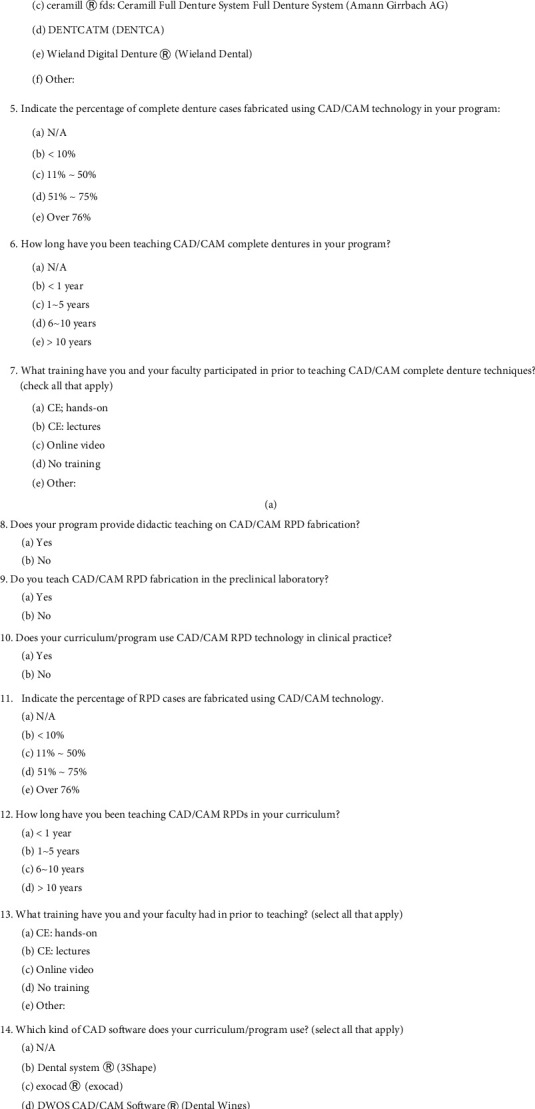
Questionnaire distributed to the directors of predoctoral prosthodontics of schools and advanced graduate prosthodontics programs of schools listed in the 2018–19 American Dental Education Association Directory.

This study was approved by the Institutional Review Board of Harvard Medical School (IRB18-0747). An online survey consisting of 15 questions was created using online survey software (Qualtrics, Qualtrics, Provo, UT, USA) (Figure 1). The survey was reviewed and validated based on feedback by faculty members involved in predoctoral and advanced graduate prosthodontic education at Harvard, Boston University, and Iwate Medical University, Schools of Dental Medicine. The survey was distributed to the directors of predoctoral prosthodontics of 56 schools and advanced graduate prosthodontics programs of 52 schools listed in the 2018–19 American Dental Education Association (ADEA) Directory. Reminders were sent in the second and fourth weeks and the second month after the initial invitation, a total of 3 reminders for 3 months to complete the survey. Participants' personal information including names was anonymized.

## 3. Results

The response rate of the predoctoral directors was 53% (26 out of 56 schools) and 52% for the advanced prosthodontic programs (27 programs out of 52). The names of the dental schools were recorded for data collection purposes only, and all survey results remained anonymous.

The percentage of programs (PP and AGP) implementing CAD-CAM complete dentures (CAD-CAM CDs) and CAD-CAM removable partial dentures (CAD-CAM RPDs) in their didactic, preclinical, and clinical curricula were recorded. CAD-CAM CD were taught in didactic courses in 54.2% of PP and 65.2% of AGP. However, CAD-CAM RPD was only taught in 37.5% of PP and 47.8% of AGP didactic curricula. Both PP and AGP, however, have implemented CAD-CAM CD and CAD-CAM RPD more in their clinical practice and didactic classes as compared with preclinical classes (Table 1). CAD-CAM CD was taught in preclinical courses in 4.2% of PP and 8.7% of AGP, while CAD-CAM RPD was taught in 12.5% of PP and 13.1% of AGP. In clinical practice, CAD-CAM CD was also utilized in 37.5% of PP and 65.2% of AGP, while CAD-CAM RPD was utilized in 25% of PP and 52.2% of AGP. Overall, AGP programs have implemented the use of CAD-CAM in all facets of their curriculum more so than PP.

### 3.1. CAD-CAM Complete Dentures

Regarding the number of years of CAD-CAM CD implementation in the curriculum, a majority of both AGP and PP answered 1∼5 years, and zero programs answered more than 10 years. Interestingly, 11.1% of PP reported having CAD-CAM CD in their clinical curriculum for 6∼10 years, which was very similar to the 12.5% reported for AGP. Both the AGP and PP programs that implemented CAD-CAM CD for 1∼5 years indicated the lowest level of implementation in preclinical laboratory exercises compared with didactic and clinical practice. Programs that implemented CAD-CAM CD less than 1 year ago indicated the highest level of implementation in preclinical laboratory exercises. The PP programs that most recently implemented CAD-CAM CD in their curricula had more emphasis in preclinical teaching (66.7%), indicating that better preclinical education materials have become available in recent years.

**Figure 2:**
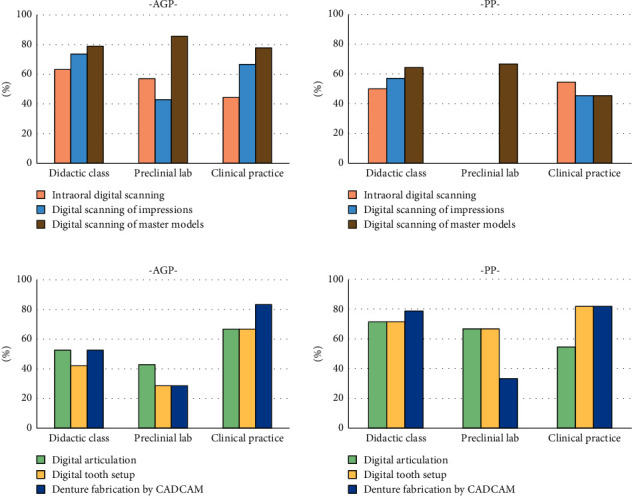
Contents of CAD-CAM complete denture teaching in didactic class, preclinical lab, and clinical practice. PP: predoctoral prosthodontics; AGP: advanced graduate prosthodontics.

When asked about the content of their CAD-CAM CD curricula, the majority of programs taught digital scanning/impressions, digital articulation, digital tooth setup, and CAD-CAM CD denture fabrication (Figure 2). In terms of impressions, digital scanning of master models was the most preferred method, and nearly 80% of AGP utilized it in clinical practice. Interestingly, none of the PP programs taught digital scanning of impressions or intraoral digital scanning in preclinical exercises. More than 50% of AGP and PP taught digital articulation, digital tooth setup, and denture fabrication using CAD-CAM techniques in didactic courses and clinical practice. Over 80% of AGP and PP used CAD-CAM denture fabrication techniques in their clinical practice. Comparing AGP and PP, AGP has implemented CAD-CAM articulation and tooth setup less in preclinical exercises.

CAD-CAM CD systems implemented in the AGP curricula included AvaDent® (87.5%), DENTCATM® (12.5%), and Wieland Digital Denture® (6.3%), and PP curricula utilized AvaDent® (66.7%) and Baltic Denture System® (8.3%) (Table 2). Regarding the percentage of cases that used CAD-CAM technology to fabricate complete dentures, 90% of PP and 80% of AGP answered <10%. 20% of AGP used CAD-CAM technology for 11∼50% of cases, indicating a higher implementation level in AGP as compared with PP. None of the programs utilized CAD-CAM technology for more than 50% of cases meaning that conventional techniques still remained the standard method (Table 2).

### 3.2. CAD-CAM Partial Denture

With regard to the number of years of implementation of CAD-CAM RPD in the curriculum, there was a similar trend as was observed with CAD-CAM CD. The majority of both AGP and PP answered that their implementation was within 1 to 5 years or less than 1 year, and no programs answered more than 10 years.

**Figure 3:**
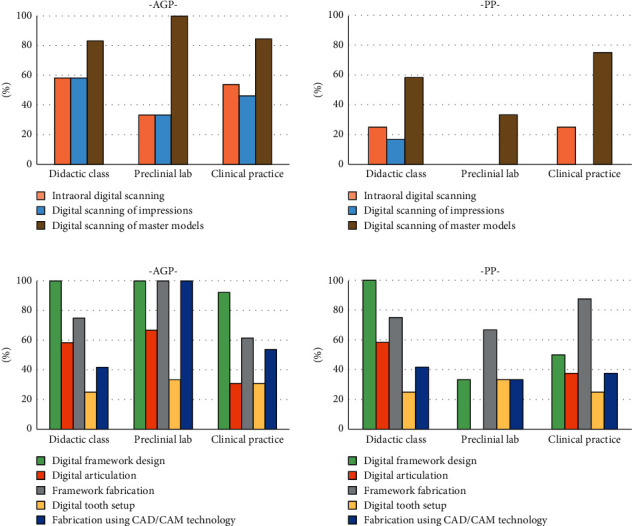
Contents of CAD-CAM partial denture teaching in didactic class, preclinical lab, and clinical practice. PP: predoctoral prosthodontics; AGP: advanced graduate prosthodontics.

When asked, “What content of CAD-CAM RPD does your program teach?” The majority were teaching digital scanning/impression, digital articulation, framework design/fabrication, digital tooth setup, and fabrication (Figure 3). For impressions, digital scanning of master models was the most preferred method for both AGP and PP in didactic, preclinical and clinical practice. The majority of AGP (92∼100%) implemented digital framework design in their didactic, preclinical, and clinical curriculum. On the contrary, 100% of PP implemented framework design and fabrication in their didactic curriculum, but only 33% and 50% in their preclinical and clinical curriculum, respectively.

The implemented CAD-CAM RPD systems in the curriculum were Dental System® (66.7%), Exocad® (28.6%), and DWOS CAD-CAM Software® (10.0%) for AGP and 50.5%, 26.3%, and 0% for PP, respectively. Other system used included coDiagnostics®, NobelBiocare®, MeshMixer®, Planscan®, and Stoneglass® (Table 3). Regarding the percentage of cases that used CAD-CAM technology to fabricate partial dentures, 37.5% of PP and 50% of AGP answered <10%, while 37.5% of PP and 33.3% of AGP answered 11∼50%. Interestingly, 25% of PP and 16.7% of AGP answered over 76% (Table 3).

### 3.3. Faculty Development of CAD-CAM Removable Prosthesis

For the preparation of the CAD-CAM CD, more AGP faculty took continuing education (CE) lectures (76.2%) and hands-on courses (57.1%) compared with PP faculty, 52.4% and 33.3%, respectively. For CAD-CAM RPD, the same trend was observed, but less faculty took CE and hands-on lectures. Approximately 38 to 45% of PP faculty did not complete any training (Table 4).

### 3.4. Challenges of Implementation of CAD-CAM Technology for the Removable Prosthodontics

There were certain challenges to the implementation of CAD-CAM technology in removable prosthodontics. The most common obstacle was the “lack of faculty and staff availability/training” in both PP (84.2%) and AGP (71.4%). For PP, “cost” (73.7%) and “time in curriculum” (78.9%) were the second largest challenges, while “resources/equipment” (61.9%) was the second largest challenge for AGP. Overall, AGP faced fewer challenges than PP in the implementation of CAD-CAM technology.

## 4. Discussion

Both in removable and fixed prosthodontics, digital technology provides multiple benefits, including improved patient experience, reduced processing errors, simplified laboratory procedures, improved communications with the dental laboratory technicians, and a more effective teaching and learning experience.

Our study indicated that more than half of the participating PP and AGP programs have implemented CAD-CAM complete dentures in their didactic courses. M. A. Fernandez et al. reported in 2015 that only 12% of schools included CAD-CAM complete dentures in their didactic curriculum, indicating an increase in the prevalence of CAD-CAM CD and CAD-CAM RPD among PP and AGP programs [18]. The increase in use of CAD-CAM could be attributed to its various advantages compared with traditional methods, including increased efficiency, accuracy, and its ability to promote interactive learning methods. Along with the overall advancements in technology, the use of CAD-CAM has been expanded in dental education. Demonstrating this, the PP programs that most recently implemented CAD-CAM CD in their curricula had more emphasis on preclinical teaching (66.7%), which may indicate that better preclinical education materials have become available in recent years. The Commission of Dental Accreditation Standards also emphasized the need for teaching the technologies both in didactic and clinical education to “support learning in different ways, including self-directed, distance and asynchronous learning” [18]. Although 37.5% of PP and 65% of AGP programs have implemented CAD-CAM CD in their clinical practice, only 10% of complete denture cases were fabricated utilizing the CAD-CAM technology, indicating that conventional methods are still predominantly used. Since the majority of schools answered that they implemented the system within the past 5 years, it will take more time for the system to be utilized routinely.

Compared to CAD-CAM CD, it was evident that fewer programs implemented CAD-CAM RPD in didactic and clinical practice. This discrepancy may be due to the inherent complexity of RPD framework design and fabrication compared to complete dentures. RPDs require uniquely complex steps such as surveying, unlike in CD fabrication. Literature on the accuracy of CAD-CAM removable prosthodontics has demonstrated greater accuracy of complete denture fabrication compared with that of partial denture fabrication [13–15]. However, both PP and AGP implemented CAD-CAM RPD in preclinical practice more than CAD-CAM CD. Specifically, 50% of programs used CAD-CAM technology for framework design and framework fabrication. The findings indicate that the technology has enabled digital framework design using scanned master models, an excellent tool for preclinical education.

Based on the results of the survey, the majority of programs reported introducing CAD-CAM technology for complete dentures in their predoctoral and advanced graduate programs. Compared with the 12% of programs in 2015 that reported having digital dentistry in their curriculum [18], our current results indicated a significant increase in the number of programs with digital dentistry in their curricula. More than half of the predoctoral programs have introduced digital dentistry into their preclinical courses, but less than a half have introduced it into their clinical training. On the contrary, postdoctoral programs have more emphasis on digital dentistry in their clinical training compared to their didactic coursework. Overall, AGP has implemented CAD-CAM RPD and CAD-CAM CD more in their curricula as compared with PP. This may be due to the difference in the breadth of information being covered at each level of education. PP programs are tasked with covering the entire breadth of removable prosthodontics from the basics of anatomy, biology, and concepts of occlusion to the foundation of CD and RPD, which becomes more challenging to students learning these topics for the first time. AGP programs, however, are delving further into advanced concepts of removable prosthodontics such as different techniques and styles of CD and RPD fabrication including a digital workflow.

It was found that the majority of schools were facing challenges in the implementation of CAD-CAM removable prostheses in their curriculum. More than 70% of PP had challenges with the cost, lack of faculty, and time available in the curriculum. AGP indicated fewer challenges compared with PP, yet 70% pointed out a lack of faculty. The shortage of faculty is an unresolved problem in dental education, and a continuous effort to promote a better understanding of the broad scope of academic life is critical [19,20]. There are always transitional periods associated with introducing new technologies due to financial constraints, workforce, and curricular time. Thus, appropriate faculty development on the technology is critical. According to Hendricson et al., most new faculty recruits are in the 55 to 60-year age range and are changing their career paths from private practice, military service, or public health positions [21]. This could pose some difficulties in adequately preparing faculty to teach this new technology. It suggests that dental schools should produce graduates who are well versed in CAD-CAM dentistry.

The annual ADEA Survey of Dental School Seniors (ADEA SDSS) in 2016 indicated that 21.4% of seniors felt under-prepared and 30.8% felt somewhat under-prepared in terms of practicing digital dentistry [22]. As the field of dental medicine continues to adopt new technologies, providers must remain current by participating in continuing education courses and analyzing relevant literature. It was reported by the ADEA SDSS that the cumulative undergraduate and dental school debt of seniors graduating from public schools rose from $102,022 in 1996 to $238,582 in 2016 (1996 currency adjusted to 2016 rates). The debt of seniors graduating from private schools rose from $179,525 in 1996 to $291,668 in 2016 [22]. Considering the high cost of dental education, incorporating new technologies into the curricula will give students a greater return on their academic investment which will ultimately improve their future practices and patient care.

Rapid technology development by companies is also a motivating factor in the implementation of CAD-CAM complete dentures with fully digitized processes including digital impression of oral soft tissue structures and digital articulation. Currently, AvaDent® provides an educational system for predoctoral prosthodontics which has been very helpful in many institutions. Collaboration between dental companies and dental schools is key for the efficient implementation of new technologies in the curricula. Continuous advancements in technology will drive dental curricula to adopt CAD-CAM and other technologies which are becoming increasingly relevant in clinical practice. Our current study serves as a robust starting point for assessing the state of CAD-CAM implementation in removable prosthodontic education. This data may be used to help guide the future standards for dental school curricula. One limitation of our current study is the response rate. 53% (26 out of 56 schools) of predoctoral directors and 52% (27 programs out of 52) of advanced graduate prosthodontic directors responded to this survey. The survey questionnaire was also limited in its choice of the CAD-CAM systems. The newly introduced CAD-CAM systems and other open-source software could be included and present useful information. While this is a sizable response rate for a survey, our conclusions may under or overestimate the current implementation level of CAD-CAM in removable prosthodontics both at the predoctoral and postdoctoral level. Further surveys on the available institutional support for the implementation of the digital technology for both fixed and removable prosthodontics and the use of open-resource software will be valuable for dental education.

## 5. Conclusions

We are amidst an age of rapidly evolving technology at large, and dentistry is no exception to this trend. New digital technologies are being developed every day and are proving to yield more accurate and efficient clinical outcomes. In order to produce clinicians that are competent in using these technologies, dental education must be at the forefront of these advancements. By assessing the current state of CAD-CAM removable prosthodontics in dental curricula, we can predict the trends in implementation and understand the barriers that prevent schools from adopting these technologies. Our current study demonstrates that digital technologies have indeed become more prevalent in dental education, but many face barriers to implementation. Programs are largely limited by a lack of funds, resources, time, and faculty members. More research must be conducted in order to support the continued incorporation of digital technologies into dental education.

## Figures and Tables

**Table 1:** Implementation of CAD-CAM denture in the curriculum.

	Complete denture		Partial denture	
	PP	AGP	PP	AGP
Didactic class	54.2%	65.2%	37.5%	47.8%
Preclinical lab exercise	4.2%	8.7%	12.5%	13.1%
Clinical practice	37.5%	65.2%	25.0%	52.2%

PP: predoctoral prosthodontics; AGP: advanced graduate prosthodontics.

**Table 2:** The characteristic of CAD-CAM CD system used, and the percentage of cases fabricated using CAD-CAM technology.

CAD-CAM CD denture system used				
AvaDent®		Wieland Digital Denture®	DENTCATM®	Baltic Denture System®
AGP	87.5%	6.3%	12.5%	0.0%
PP	66.7%	0.0%	0.0%	8.3%
The percentage of complete denture cases fabricated using CAD-CAM technology				

	<10%	11%∼50%	51%∼75%	>76%
AGP	81.3%	18.8%	0.0%	0.0%
PP	90.0%	10.0%	0.0%	0.0%

PP: predoctoral prosthodontics; AGP: advanced graduate prosthodontics.

**Table 3:** Characteristics of the CAD-CAM RPD system used and frequency.

CAD-CAM RPD system used				
Dental System® (3Shape)		Exocad® (excad)	DWOS CAD-CAM Software® (dental wings)	Others
AGP	66.7%	28.6%	19.0%	28.6%
PP	50.5%	26.3%	0%	23.2%
The percentage of RPD cases fabricated using CAD-CAM technology				

	<10%	11% ∼ 50%	51% ∼ 75%	>76%
AGP	50.0%	33.3%	0.0%	16.7%
PP	37.5%	37.5%	0.0%	25.0%

PP: predoctoral prosthodontics; AGP: advanced graduate prosthodontics.

**Table 4:** The training faculty have had prior to teaching (checked all that apply).

Contents	CAD-CAM CD		CAD-CAM RPD	
	PP (%)	AGP (%)	PP (%)	AGP (%)
Continuing education: lectures	52.40	76.20	38.90	45.50
Continuing education: hands-on	33.30	57.10	27.80	45.50
Online video	38.10	33.30	33.30	18.20
No training	38.10	19.00	44.40	36.40

PP: predoctoral prosthodontics; AGP: advanced graduate prosthodontics.

## Data Availability

The data used to support the findings of this study are available from the corresponding author upon request.
